# Sex hormone supplementation improves breathing and restores respiratory neuroplasticity following C2 hemisection in rats

**DOI:** 10.3389/fphys.2024.1390777

**Published:** 2024-05-13

**Authors:** Rebecca Barok, Jessica M. L. Grittner, Shawn Miller, Brendan J. Dougherty

**Affiliations:** ^1^ Rehabilitation Science Graduate Program, Department of Family Medicine and Community Health, University of Minnesota Medical School, Minneapolis, MN, United States; ^2^ Division of Physical Therapy and Rehabilitation Science, Department of Family Medicine and Community Health, University of Minnesota Medical School, Minneapolis, MN, United States

**Keywords:** spinal cord injury, testosterone, estrogen, phrenic, plasticity

## Abstract

In addition to loss of sensory and motor function below the level of the lesion, traumatic spinal cord injury (SCI) may reduce circulating steroid hormones that are necessary for maintaining normal physiological function for extended time periods. For men, who comprise nearly 80% of new SCI cases each year, testosterone is the most abundant circulating sex steroid. SCI often results in significantly reduced testosterone production and may result in chronic low testosterone levels. Testosterone plays a role in respiratory function and the expression of respiratory neuroplasticity. When testosterone levels are low, young adult male rats are unable to express phrenic long-term facilitation (pLTF), an inducible form of respiratory neuroplasticity invoked by acute, intermittent hypoxia (AIH). However, testosterone replacement can restore this respiratory neuroplasticity. Complicating the interpretation of this finding is that testosterone may exert its influence in three possible ways: 1) directly through androgen receptor (AR) activation, 2) through conversion to dihydrotestosterone (DHT) by way of the enzyme 5α-reductase, or 3) through conversion to 17β-estradiol (E2) by way of the enzyme aromatase. DHT signals via AR activation similar to testosterone, but with higher affinity, while E2 activates local estrogen receptors. Evidence to date supports the idea that exogenous testosterone supplementation exerts its influence through estrogen receptor signaling under conditions of low circulating testosterone. Here we explored both recovery of breathing function (measured with whole body barometric plethysmography) and the expression of AIH-induced pLTF in male rats following C2-hemisection SCI. One week post injury, rats were supplemented with either E2 or DHT for 7 days. We hypothesized that E2 would enhance ventilation and reveal pLTF following AIH in SCI rats. To our surprise, though E2 did beneficially impact overall breathing recovery following C2-hemisection, both E2 supplementation and DHT restored the expression of AIH-induced pLTF 2 weeks post-SCI.

## 1 Introduction

According to the most recent data from the National Spinal Cord Injury Database, nearly 300,000 Americans are living with spinal cord injury (SCI), with roughly 18,000 new SCI occurring annually (National Spinal Card Injury Statistical Centre, 2022). The incidence of SCI leans heavily male, as 78% of new SCIs occur in males, a statistic that has remained steady since national statistics have been collected (National Spinal Card Injury Statistical Centre, 2022). SCI impairs motor and sensory function below the level of injury. Since half of all SCI’s occur at cervical spinal cord levels (National Spinal Card Injury Statistical Centre, 2022), motor dysfunction to upper and lower extremities (i.e., tetraplegia) is common. Further, phrenic motor neurons controlling the diaphragm muscle are located in the ventral horn of cervical spinal cord segments. Because of this anatomical location, individuals experiencing cervical SCI will often experience some level of respiratory compromise (Gonzalez-Rothi et al., 2015). These respiratory issues may reduce functional mobility and exercise tolerance, or at worse, may necessitate mechanical ventilation for survival. Respiratory related complications, such as pneumonia, are one of the principal causes of mortality in the SCI population (National Spinal Card Injury Statistical Centre, 2022). Thus, research aimed at strengthening respiratory motor function is of high priority.

In addition to primary and secondary tissue damage associated with traumatic SCI, injury-induced hormone dysregulation in both females and males is frequently of chronic concern. In females, disruption of regular menstrual cycles and potential dysregulation of circulating hormones like 17β-estradiol (E2) can last for months following SCI (Sipski, 1991; Huang et al., 1996; Charls et al., 2022). In males, testosterone, the principle circulating sex steroid hormone, is systemically reduced in nearly all men with SCI for the first 12-month (Clark et al., 2008; Yarrow et al., 2020). Lifelong reductions in testosterone are estimated in 50% of males with SCI and low testosterone is associated with reduced muscle mass, and reduced bone density (Yarrow et al., 2014; Otzel et al., 2018). E2 and testosterone are also known to have anti-inflammatory characteristics (Sribnick et al., 2005; Samantaray et al., 2010; Lara et al., 2021). Thus, injury-induced reductions in these hormones may contribute to increased secondary damage and subsequent functional deficits (Tanzer and Jones, 1997; Huppenbauer et al., 2005). Although the exact etiology of SCI-induced hormone dysregulation is unknown, and likely multifactorial, one possible contributing factor involves trauma-induced central suppression of the hypothalamic-pituitary-gonadal axis. Injury-induced stress and/or inflammation can cause decreased production of luteinizing and follicle stimulating hormones that are necessary precursors for gonadal hormone production (Sullivan et al., 2017; Nedresky and Singh, 2024). To ameliorate physiological comorbidities related to diminished hormone availability, exogenous steroid hormones have been experimentally utilized to enhance functional recovery following SCI in pre-clinical and clinical studies (Byers et al., 2012; Yarrow et al., 2014; Bielecki et al., 2016; Yarrow et al., 2017; Otzel et al., 2018; Yarrow et al., 2020). Yet, the effects of hormone supplementation on restoration of breathing function following SCI is unknown.

**FIGURE 1:**
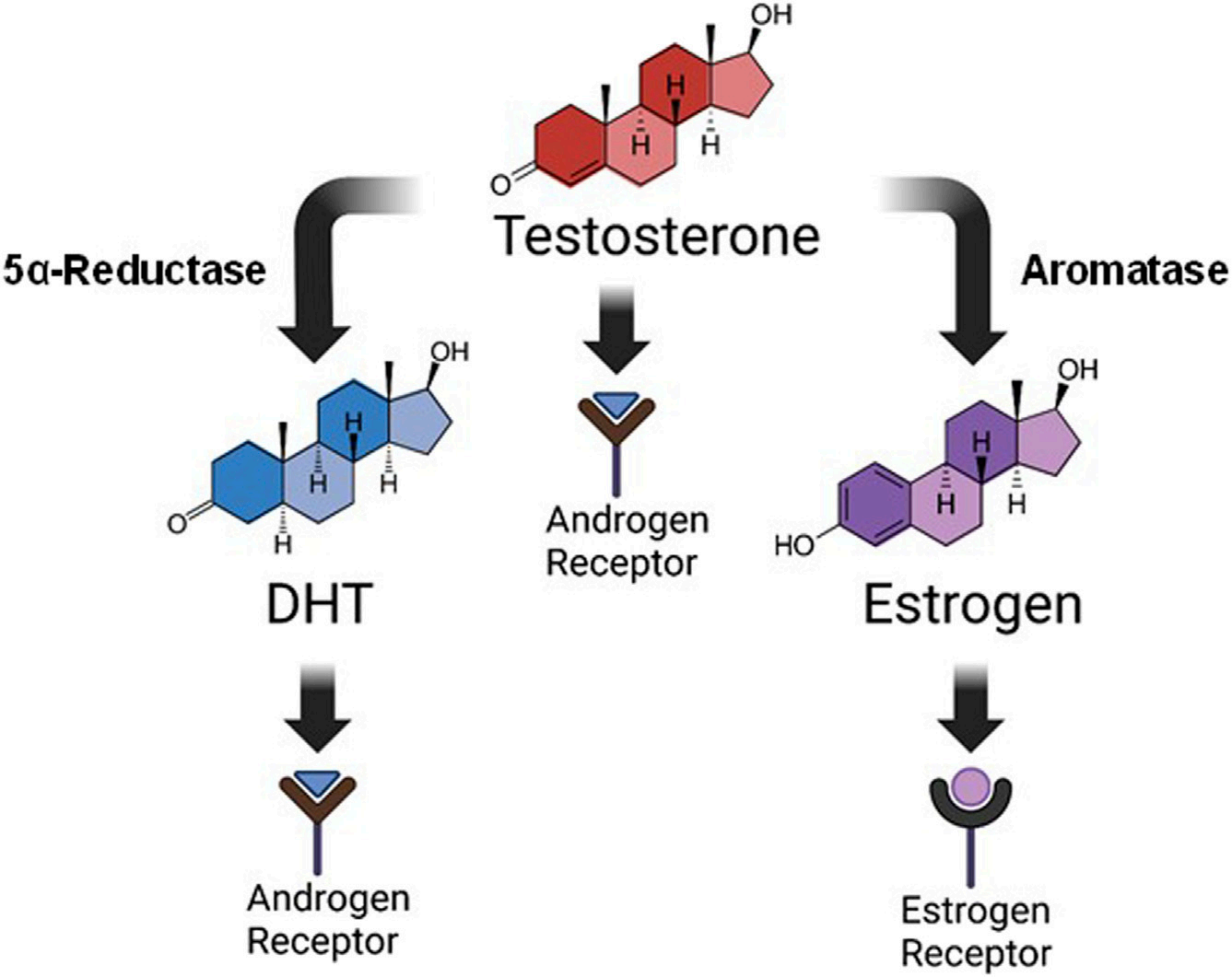
Testosterone metabolism. Steroid hormones are derived from the breakdown of cholesterol via multiple metabolizing enzymes. This image is a simplified rendition showing that testosterone, derived ultimately from cholesterol metabolism, can influence physiological function in three distinct ways: 1) direct activation of androgen receptors, 2) conversion to dihydrotestosterone (DHT) by 5α-reductase where it also signals through androgen receptors, or 3) conversion to estrogen (specifically 17β-estradiol; E2) by aromatase, and subsequent activation of estrogen receptors. Our study focused on supplementation of DHT or E2 for improving respiratory function following C2-hemisection.

Steroid hormones are known to play a key role in respiratory motor function, and in the expression of respiratory neuroplasticity. For example, young adult female rats only express long-term facilitation of phrenic neural activity (pLTF) in response to acute intermittent hypoxia (AIH) during stages of the estrous cycle where E2 levels are elevated (Dougherty et al., 2017). These effects appear to be independent of hypoxic sensitivity, as the neural response to hypoxia is similar in female rats regardless of E2 availability (Marques et al., 2017; McIntosh and Dougherty, 2019; Barok et al., 2021). Removal of the ovaries and subsequent loss of circulating E2 also eliminates AIH-induced pLTF in young female rats, an effect that can be rescued with systemic supplementation, or spinal application of E2 (Dougherty et al., 2017). In males, testosterone plays a role in respiratory function and the expression of respiratory neuroplasticity. Young adult male rats are unable to express AIH-induced pLTF following castration, an effect that is reversed with systemic testosterone supplementation (Zabka et al., 2006). Complicating the interpretation of this finding is that testosterone may exert its influence in three possible ways: 1) directly through androgen receptor (AR) activation, 2) through conversion to dihydrotestosterone (DHT) by way of the enzyme 5α-reductase, or 3) through conversion to E2 by way of the enzyme aromatase. DHT signals via AR activation similar to testosterone, but with higher affinity, while E2 activates local estrogen receptors (Figure 1). In castrated males, DHT did not restore pLTF nor did testosterone supplementation when enzymatic aromatase activity was blocked (Zabka et al., 2006). Thus, evidence to date supports the idea that exogenous testosterone supplementation may exert its influence through estrogen receptors under conditions of low circulating testosterone.

Here we explored the role of DHT and E2 to both recovery of breathing function (measured with whole body barometric plethysmography) and the expression of AIH-induced pLTF in male rats following C2-hemisection (C2Hx) SCI. In a preliminary cohort of male rats, we established that testosterone levels declined following C2Hx, similar to what has been documented in humans. In our experimental cohort, intact (sham surgery) rats were compared to rats with a SCI, with or without hormone supplementation. One week post injury, rats were supplemented with either E2, or DHT (the non-aromatizable form of testosterone that acts via AR activation) for 7 days. Data collection was 2-weeks following injury, when ipsilateral phrenic neural activity often reemerges (Lane et al., 2009), but pLTF is known to be depressed (Golder and Mitchell, 2005). Although prior studies indicate that hormone supplementation immediately following injury offers unique neuroprotective characteristics that benefit functional recovery (Sengelaub et al., 2018), we delayed our treatment to explore impacts specifically in the sub-acute period of recovery. We hypothesized that E2 would enhance ventilation and reveal pLTF following AIH since prior work suggested that conversion of testosterone to E2 was important for neuroplasticity in males. Our results showed that E2 beneficially impacted breathing recovery following C2Hx as hypothesized. However, both E2 supplementation and DHT restored the expression of AIH-induced pLTF 2 weeks post-SCI.

## 2 Materials and methods

All experimental protocols were approved by the Institutional Animal Care and Use Committee at the University of Minnesota. A total of 28 Male Sprague Dawley rats (3–4 mos) were obtained from Envigo Colony 206 and housed in an AAALAC accredited animal facility. Rats were housed in pairs with *ad libitum* access to food and water in a 12:12 h light-dark cycle (on 0600–1800). All rats were acclimated to the facility for a minimum of 1 week prior to experimental manipulation and were randomly assigned to one of four experimental groups: sham (laminectomy surgery), SCI-placebo, SCI-E2, or SCI-DHT. All groups (*n* = 7 each) were age matched at times of data collection. An additional 10 rats were used in preliminary studies to explore the impact of SCI on serum testosterone levels.

### 2.1 Spinal cord injury surgeries

Buprenorphine SR (1 mg/kg, s.c.) was given to each rat at least 2 h before the start of surgery for long-lasting pain management. Rats were briefly anesthetized in an induction chamber with 5% isoflurane in 100% O_2_, then placed prone on a surgery table where 2%–4% isoflurane anesthesia was maintained via nosecone. Adequate depth of anesthesia was assessed by regularly testing toe pinch and eye blink reflexes. A two-inch section of the upper back, from the base of the skull, was shaved and cleaned with chlorohexidine. After palpation of the cervical vertebrae, a midline incision was made from the base of the skull to approximately C7. The overlying muscles were cut and retracted exposing the C2 vertebrae. Using bone rongeurs, ∼90% of the C2 lamina was removed exposing the dorsal spinal cord. With a surgical microscope, the dorsal spinal roots at segment C3 were identified, and a microscalpel or a 22-gauge needle was inserted at the spinal midline just rostral to the C3 rootlets and swept laterally to induce a left C2 hemisection. The scalpel or needle was reinserted to confirm lesion completeness to the full ventral depth. After hemisection, a small piece of Durafilm (FEP, American Durafilm, Holliston, MA) was placed over the spinal cord incision site to minimize muscular scar adhesion to the spinal cord. Overlying muscles were sutured with 4–0 dissolvable sutures (Covidien, Mansfield, MA) and the skin closed using 9 mm skin clips (Braintree Scientific, Gardena, CA). Rats were removed from isoflurane and relocated to a heated recovery cage where they recovered under laboratory supervision for 72 h. Daily post-op care included fluid and nutrition supplementation, antibiotics, bladder expression if needed (this was rare and temporary), and pain management with buprenorphine. After 72 h, rats were returned to the animal housing facility and given 1 week to recover. All SCI surgeries were completed by an experienced surgeon who was blinded to SCI group designation. Rats in the sham group underwent identical surgical procedures and received identical post-operative care, however, the spinal cord was left intact.

### 2.2 Testosterone ELISA

Preliminary studies were carried out to determine if C2Hx reduced circulating testosterone levels 2 weeks post-SCI in 10 male rats. Blood samples were collected directly from the left ventricle prior to transcardial perfusion. Blood was placed in a 4°C fridge overnight for coagulation, then centrifuged at 9000 RPM for 15 min to separate serum from red blood cells. Serum was collected and stored at −20°C until the morning of enzyme-linked immunosorbent assay (ELISA) testing. Testosterone ELISA was completed per manufacturer’s instructions (DRG International, Springfield, NJ). Analysis was completed following creation of a standard curve from provided control samples. Sensitivity of the ELISA was 0.083 ng/mL with a range of 0.083–16 ng/mL.

### 2.3 Time-release hormone pellet implantation

One week following C2Hx surgery, and coinciding with the removal of SCI skin clips, rats in the SCI-E2, SCI-DHT, and SCI-placebo groups received time-release hormone pellet implants. Two hours prior to the procedure, rats again received s.c. buprenorphine SR (1 mg/kg) for pain management. Isoflurane induction was briefly carried out in a closed container and maintained via nose cone at 2%–4% in 100% O_2_. Depth of anesthesia was confirmed by lack of observable toe pinch and eye blink reflexes. A small area of dorsal skin between the right shoulder blade and right ear was shaved and cleaned with chlorohexidine. A 5–8 mm incision was made through the skin and using blunt dissection of fascia, a pocket was formed under the skin between the shoulder blade and the neck. A small time-release drug pellet (Innovative Research of America, Sarasota, FL) containing either 17β-estradiol 3 benzoate (E2; 10 µg/day), 5α dihydrotestosterone (DHT; 50 µg/day), or a placebo pellet, was inserted into the pocket and the incision was closed using a single 9 mm wound clip. The dose of DHT is on the lower end of the range known to produce plasma titers in the physiological range in adult male rats (Sengelaub et al., 2018). E2 dosing was administered to maintain physiological levels similar to those measured in female rats during proestrus (Dougherty et al., 2017). Rats recovered quickly in a heated recovery chamber before being returned to their home cages. Daily care was administered as needed for 72 h in collaboration with UMN Research Animal Services. Pellet implant procedures took <10 min.

### 2.4 Whole body plethysmography

Barometric, whole-body plethysmography (Data Science International, St. Paul, MN) was used to measure ventilation in normoxia and under hypoxic-hypercapnic conditions. Data was collected 7 days after pellet implantation for the SCI-E2, SCI-DHT, and SCI-placebo groups (2 weeks post-SCI or sham surgery). This time point was chosen to capture recovery of breathing function during the sub-acute recovery period. After obtaining core body temperature via rectal probe, rats were placed individually into clear Plexiglas containers (4.0-L, Data Science International, St. Paul, MN). A customizable gas mixer (GSM-3, CWE, Inc., Ardmore, PA) delivered inspired gas to each chamber at a rate of 2.5 L of gas per minute, allowing for easy control of inspired gas concentrations. All ventilation data was collected using customizable data acquisition software (Ponemah, Data Science International, St. Paul, MN) from each chamber in real time. The rats were exposed to an hour of normoxia (O_2_—20.9%, N_2_ balance; Baseline, BL), followed by a 10-min respiratory challenge (O_2_—10%, CO_2_—5%, N_2_ balance, Max). Chamber temperature, barometric pressure, and humidity were measured constantly throughout the experiment and used in the calculations of respiratory variables. At the conclusion of the respiratory challenge period, rats were promptly removed from the chamber and rectal temperatures were taken again. All plethysmography tests were administered at the same time of day, 4–6 h into the light phase of a 12-h light/dark cycle.

Respiratory frequency (ƒ), was derived from air pressure traces that were continuously sampled at 500 Hz. Tidal volume (VT) and minute ventilation (VE) were calculated by the Ponemah software and are presented as raw values and normalized for weight. An independent data analyzer, blinded to treatment, selected 5 minutes of stable breathing (minimal movement artifacts or augmented breaths) near the end of the BL period for analysis. Rats were always in an apparent state of rest or sleep (lying in curled position, eyes closed) during BL data collection. All rats were aroused during the hypoxic-hypercapnic challenge, sitting in the chamber on all fours in a position of focused breathing. Following stabilization of the chamber gas mixture, a 2-min segment of stable breathing in the latter third of Max challenge was used for analysis.

Expired gas from each chamber was sampled at a rate of 300 mL/min using subsampler pumps (Sable Systems, North Las Vegas, NV). A third sample was taken via subsampler from the inspired gas line prior to entering the chambers. The subsamplers connected to a multiplexor unit (Sable Systems, North Las Vegas, NV) that diverted samples from each chamber (and the inspired gas) to an oxygen/carbon dioxide analyzer (Gemini, CWE, Ardmore, PA) every 30 s. O_2_ and CO_2_ measures were linked to Ponemah for real-time calculation of oxygen utilization (VO_2_) and carbon dioxide production (VCO_2_) in each rat. Since differences in BL VCO_2_ were observed, and minute ventilation can be strongly influenced by VCO_2_, we calculated VE relative to VCO_2_ in the presentation of our ventilation data and included calculations of respiratory quotient (RQ; VCO_2_/VO_2_).

### 2.5 Neurophysiological preparation

Phrenic nerve recordings were collected 1–2 days following plethysmography (2 weeks post-SCI). Rats were anaesthetized initially by inhalation of isoflurane, tracheotomized and mechanically ventilated (RoVent; Kent Scientific, Torrington, CT, United States; tidal volume, 1.5–2.5 mL; frequency, 75 breaths min^−1^). Anesthesia was maintained with isoflurane (SomnoSuite; Kent Scientific; 3–4% in 50% O_2_, balance N_2_) for the duration of the surgical procedures, and the adequacy of general anesthesia was confirmed by lack of response to toe pinch and eye‐blink reflexes. Rats were slowly converted to urethane anesthesia (1.8 mg kg^−1^) via a right femoral vein catheter. Surgery was performed on a temperature‐controlled stainless‐steel surgical table. Rectal temperature was monitored continuously with a temperature sensor (RightTemp; Kent Scientific) and maintained by adjusting the temperature of the surgical table. The concentration of inspired O_2_ was monitored throughout all experiments using a fuel‐cell O_2_ sensor (AII 3000A; Analytical Industries, Pomona, CA, United States). Rats were vagotomized, and a catheter was inserted into the right femoral artery to monitor blood pressure using a calibrated pressure transducer (SP844; MEMScap, Isere, France). Blood samples obtained from the femoral artery were analyzed for partial pressures of O_2_ (PaO_2_) and CO_2_ (PaCO_2_) and pH with a blood gas analyzer (CCA‐TS2; OPTI Medical, Roswell, GA, United States); standard base excess, calculated by the analyzer, was also used as an indicator of metabolic acid–base disturbances. A slow, continuous infusion of a 5:1 mix of veterinary lactated Ringer solution and sodium bicarbonate was maintained via the femoral vein catheter after conversion to urethane anesthesia to maintain blood pressure and acid–base balance throughout the experiment. The left phrenic nerve (ipsilateral to C2-hemisection) was dissected and exposed via a dorsal approach, cut distally, and desheathed. The nerve was submerged in mineral oil and placed on bipolar silver recording electrodes to record spontaneous neural activity. The adequacy of anesthesia was tested before protocols commenced and immediately after the protocol was completed. The adequacy of anesthetic depth was assessed as the lack of pressor or respiratory neural response to a toe pinch. We did not observe increased blood pressure or respiratory nerve activity in any of the rats after toe pinch. Neuromuscular block with pancuronium bromide (2.5 mg mL^−1^) was initiated after confirmation of adequate anesthesia, in order to remove excessive movement artifacts associated with respiratory muscle activity. Instantaneous end‐tidal CO_2_ was monitored from the expired line (CapnoScan; Kent Scientific) and maintained at ∼45 mmHg throughout the surgery to allow stabilization of the preparation and initial nerve signals. Nerve activity was amplified (gain, 100,00×; A‐M systems 1800, Sequim, WA, United States), bandpass filtered (300 Hz–10 kHz), rectified and integrated (time constant, 50 ms). Resulting signals were digitized, recorded and analyzed with PowerLab (LabChart v.8 software, AD Instruments, Colorado Springs, CO, United States).

### 2.6 Neurophysiological protocol

Stable nerve activity was established while the rat was ventilated with a hyperoxic inspired gas mixture [fraction of inspired oxygen (FIO_2_), 0.5–0.6; PaO_2_, >150 mmHg], with sufficient levels of inspired CO_2_ to maintain constant arterial PaCO_2_, preventing the rat from becoming apneic (typically between 40 and 45 mmHg). After the preparation was stabilized, the apneic threshold for rhythmic phrenic activity was determined by progressively lowering the inspired (and arterial) CO_2_ until rhythmic phrenic activity ceased. From apnea, the end‐tidal CO_2_ was increased progressively every ∼90 s until nerve activity resumed (i.e., the recruitment threshold). Baseline nerve activity was established with end‐tidal CO_2_ set 2 mmHg above the CO_2_ recruitment threshold. This procedure allows a standardized level of respiratory drive during baseline conditions in different rats. A baseline blood sample was taken, and the rats were exposed to AIH, consisting of three 5 min hypoxic episodes (FIO_2_, 0.10–0.12; PaO_2_, 35–50 mmHg) interspersed with 5 min intervals of baseline conditions (i.e., FIO_2_, 0.5–0.6). Data were collected from the first hypoxic episode when possible. However, if PaO_2_ or PaCO_2_ fell out of *a priori* ranges during hypoxia, the data were gathered from subsequent hypoxic episodes. Nerve activity was monitored for 60 min after the last hypoxic episode while maintaining baseline levels of arterial blood gases. Blood samples (0.2 mL in a heparinized syringe) were obtained and analyzed before hypoxic challenge (baseline), during hypoxic challenge, and at 15, 30 and 60 min post‐hypoxia. At the conclusion of each experiment, rats were euthanized either by urethane overdose administered via the femoral vein followed by discontinuation of pump ventilation, or via transcardial perfusion with ice-cold PBS and 4% paraformaldyhyde.

### 2.7 Statistical analyses

All statistical analyses were completed using GraphPad Prism statistical software (La Jolla, CA). One-way ANOVA was used to compare age, weight, and temperature between groups for plethysmography. Ventilation data were analyzed for between-group differences and time-based changes following AIH using two-way ANOVA with repeated measures. Bonferroni *post hoc* tests were employed when main effects or interactions were present.

Peak amplitude and burst frequency of phrenic nerve recordings were averaged in 1-min bins at baseline, during hypoxic exposure, and 15, 30, and 60 min after the last hypoxic episode. Burst frequency was analyzed in bursts per minute; nerve amplitudes are reported as % change relative to BL measures. Two-way ANOVA with repeated measures was used to analyze group differences for time (e.g., baseline and 15, 30, and 60 min post-AIH) and treatment effects. One-way ANOVA was used to investigate between-group differences in baseline phrenic amplitude, phrenic nerve burst responses to hypoxia, and physiological parameters during phrenic nerve recordings. Data are presented as means ± SEM and significance was set at *p* < 0.05.

## 3 Results

### 3.1 C2-hemisection reduces circulating testosterone levels

**FIGURE 2:**
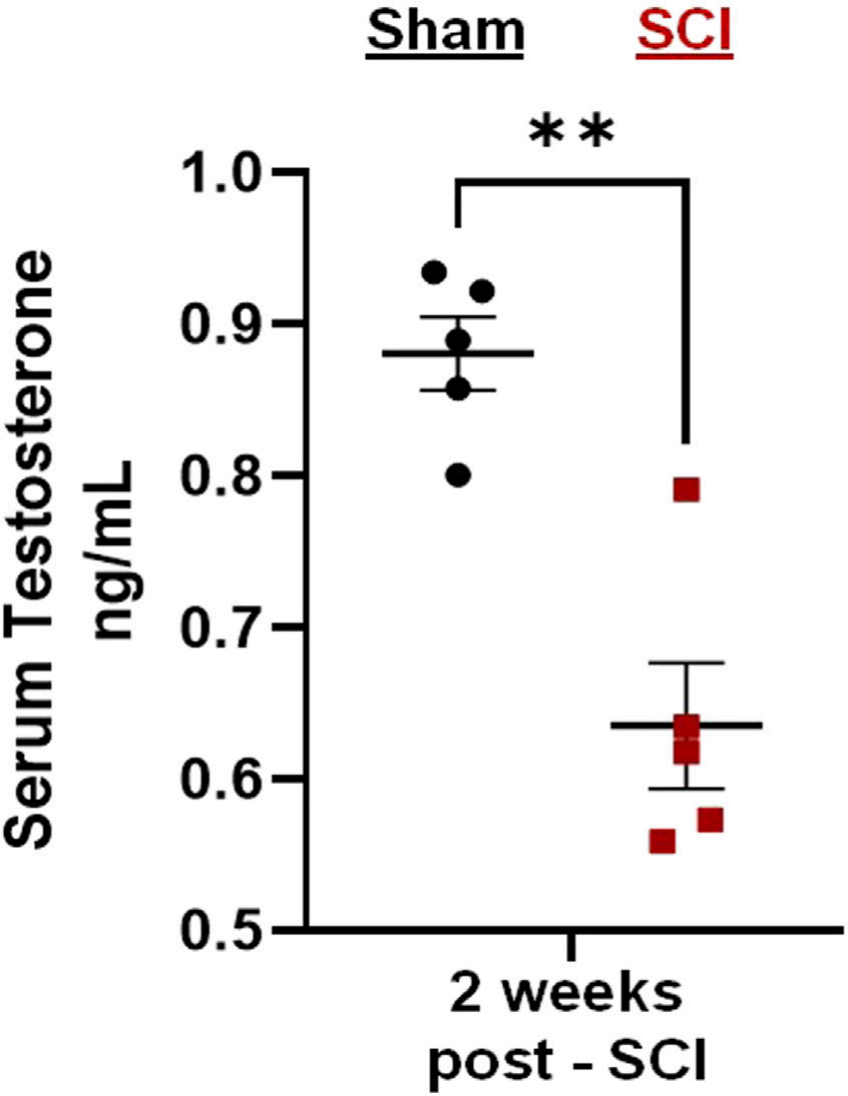
C2-hemisection reduces serum testosterone levels. C2-hemisection induces a reduction in circulating testosterone levels for at least 2 weeks post-SCI. In preliminary studies, rats received C2-hemisection or Sham surgeries (laminectomy) to determine how the injury would impact serum testosterone levels. Using ELISA, SCI rats (0.64 ng/mL) showed significantly reduced serum testosterone 2 weeks post-injury compared to Shams (0.88 ng/mL). All data were normally distributed (*p* > 0.05 for both Shapiro-Wilk test and Kolmogorov-Smirnov test) and represent mean ± SEM. Two-tailed *t*-test with Welch’s correction; **p = 0.002.

Preliminary tests demonstrated that C2Hx caused reduced levels of circulating testosterone 2 weeks post-injury (Figure 2). Serum extracted from male rats 2 weeks post-injury showed significant reductions in circulating testosterone compared to age-matched, sham rats receiving laminectomy surgeries (Figure 2; two-tailed *t*-test; *p* = 0.002).

### 3.2 17β-estradiol supplementation improves respiratory pattern in sub-acute cervical SCI

Experimental groups were of similar statistical age and body weight during barometric plethysmography. However, DHT rats tended to be younger (96 days ± 6) and lighter in weight (328 g ± 10) relative to other groups. Rectal temperatures at both the start and completion of plethysmography were comparable and temperatures did not significantly fluctuate with testing. Physiological data are shown in Table 1.

**TABLE 1:** Physiological parameters and ventilatory data in normoxia and hypoxic-hypercapnic challenge (Max).

		Sham	Placebo	SCI + DHT	SCI + E2
Age (days)		112 ± 4	124 ± 7	96 ± 6	123 ± 16
Weight (g)		377 ± 13	371 ± 19	328 ± 10	339 ± 13
Pre-Temp (°C)		37 ± 0.2	37 ± 0.3	37 ± 0.2	37 ± 0.1
Post-Temp (°C)		37 ± 0.1	37 ± 0.2	36 ± 0.1	37 ± 0.2
Baseline					
	ƒ (breaths/min)	63 ± 5.2	83 ± 8.0	71 ± 4.0	61 ± 4.6
	V_T_ (ml/breath/100 g)	0.6 ± 0.1	0.5 ± 0.1	0.6 ± 0.0	0.7 ± 0.1
	V_E_ (ml/min/100 g)	38 ± 2.1	45 ± 4.5	43 ± 2.1	39 ± 1.8
	VCO_2_ (ml/min/100 g)	2.0 ± 0.1	2.2 ± 0.2	2.3 ± 0.2	1.6 ± 0.1^#†^
Max					
	ƒ (breaths/min)	143 ± 6.7^+^	153 ± 8.7^+^	135 ± 6.7^+^	126 ± 5.3^+#^
	V_T_ (ml/breath/100 g)	1.0 ± 0.1^+^	0.9 ± 0.1^+^	0.9 ± 0.1^+^	1.1 ± 0.1^+#^
	V_E_ (ml/min/100 g)	145 ± 9.8^+^	135 ± 13^+^	128 ± 11^+^	139 ± 8.3^+^
	VCO_2_ (ml/min/100 g)	1.8 ± 0.1	1.8 ± 0.1	1.5 ± 0.3	1.3 ± 0.2

Group age, weight and temperatures were similar during barometric plethysmography testing. Two-way ANOVA, with repeated measures demonstrated that C2Hx rats receiving E2 supplementation had reduced BL VCO_2_ compared to SCI (i.e., Placebo) rats and DHT groups. All rats had increased ventilatory parameters during Max challenge relative to BL. SCI-E2 rats also showed higher Max frequency and VT compared to SCI rats. #: *p* < 0.05 from SCI-placebo rats; +: *p* < 0.05 from BL; †: *p* < 0.05 from SCI-DHT.

**FIGURE 3:**
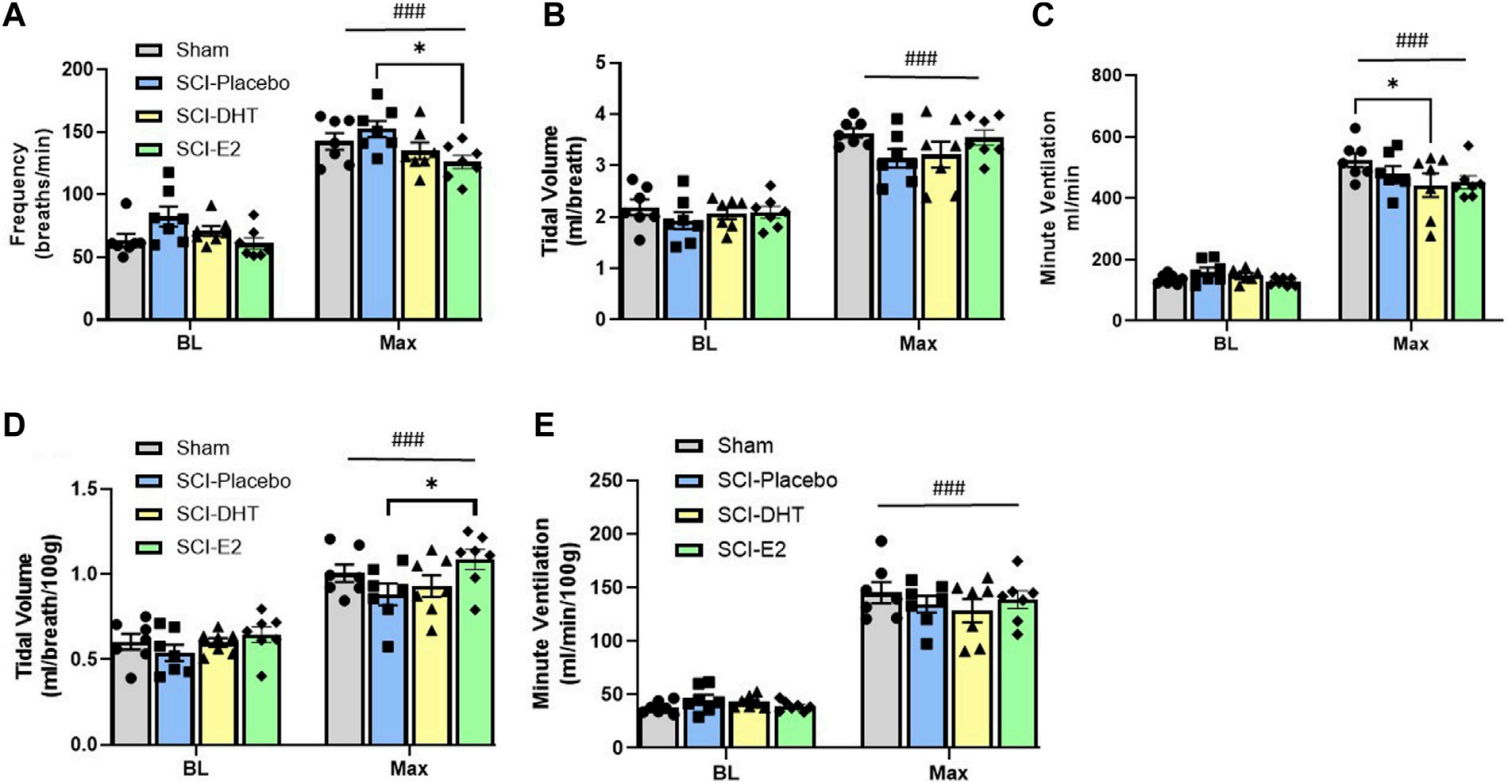
Estrogen supplementation improves ventilation following C2-hemisection. Whole body barometric plethysmography was used to measure ventilation in awake, freely-behaving rats 2 weeks post-SCI during baseline (BL) normoxia and respiratory challenge (Max). Rats receiving SCI alone (Placebo) tended to show increased respiratory frequency during BL compared to Sham rats, but this difference did not reach statistical significance **(A)**. All rats significantly increased their respiratory frequency during Max challenge **(A)**. Rats with E2 supplementation showed significantly lower frequency during Max compared with Placebo **(A)**. Raw tidal volume (VT) production was similar across groups during BL and all groups responded to Max with significantly increased VT **(B)**. Raw minute ventilation (VE) was also statistically similar during BL and significantly increased with Max in all groups **(C)**. Rats receiving DHT supplementation showed significantly reduced raw VE relative to Sham rats during Max **(C)**. VT **(D)** and VE **(E)** were also assessed relative to body weight. All rats continued to show significantly increased VT during Max, but when weight was considered, rats receiving E2 showed significantly higher VT relative to Placebo rats **(D)**. The VE response to Max challenge was similar with weight adjusted calculations, but DHT rats were no longer different than Sham rats during Max **(E)** All data assessed using two-way ANOVA with repeated measures and Bonferroni *post hoc* tests where appropriate. ###:*p* < 0.0001 vs BL; *p < 0.05.

Baseline respiratory frequency was statistically similar across experimental groups, though rats in the SCI-placebo group showed modestly elevated frequency (83 ± 8 breaths/min) compared to both uninjured Sham rats (63 ± 5.2 breaths/min; *p* = 0.18) and SCI-E2 rats (61 ± 4.6 breaths/min; *p* = 0.09; Figure 3A). A compensatory increase in BL respiratory rate is often seen in rats 2 weeks post C2-hemisection (Fuller et al., 2008; Dougherty et al., 2016; Dougherty et al., 2018). In response to Max respiratory challenge (consisting of 10% O_2_, 5% CO_2_, N_2_ balance), all groups significantly increased breathing frequency (main condition effect; *p* < 0.0001; Figure 3A). In addition, a main effect of treatment (*p* = 0.03) was observed with Max challenge; E2 supplementation significantly reduced respiratory frequency compared to SCI-placebo rats (*p* = 0.02; Figure 3A). C2Hx did not significantly impair BL tidal volume generation. Rats in the SCI-placebo group (1.93 mL/breath) generated similar BL VT as Sham rats (2.19 mL/breath; *p* > 0.90) and hormone treatment with DHT (2.07 mL/breath) or E2 (2.09 mL/breath) had no effect on raw BL VT (*p* > 0.9; Figure 3B). As with frequency, all rats significantly increased VT in response to Max challenge (main condition effect; *p* < 0.0001; Figure 3B), but no between group VT differences were observed; all groups generated similar VT during Max (Figure 3B). Values of raw VE were also similar across all experimental groups during BL (Figure 3C). VE significantly increased with Max challenge in all groups (main condition effect; *p* < 0.0001; Figure 3C) and a main effect of treatment emerged with DHT rats (442.8 mL/min) showing significantly reduced VE compared with Sham rats (524.4 mL/min; *p* = 0.04; Figure 3C) during Max.

Tidal volume and minute ventilation were also assessed relative to body weight to account for potential impacts of small weight differences between experimental groups. BL VT values were comparable across all groups (*p* > 0.89; Figure 3D) and all groups showed a significant elevation in VT generation in response to Max challenge (main condition effect; *p* < 0.0001; Figure 3D). However, rats receiving E2 showed significantly higher VT (1.09 mL/breath/100 g) during Max than SCI-placebo rats (0.88 mL/breath/100 g; *p* = 0.044; Figure 3D). VE was appropriately elevated in response to Max challenge in all groups (main condition effect; *p* < 0.0001; Figure 3E), but no differences in VE were seen between groups in BL or during Max challenge when normalized to body weight (Figure 3E). Since rats receiving DHT tended to be lighter than other groups (though not statistically different; Table 1), normalizing to body weight may more accurately reflect comparative VT and VE in response to Max challenge. All weight-normalized ventilatory data is provided in Table 1. Collectively, our findings suggest that E2 supplementation lowered respiratory frequency and increased VT production in response to Max respiratory challenge relative to untreated SCI rats.

**FIGURE 4:**
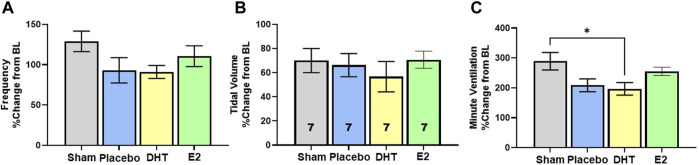
DHT supplementation reduced overall ventilation with respiratory challenge. Respiratory frequency **(A)**, tidal volume **(B)** and minute ventilation **(C)** were calculated as a %change from BL to assess magnitude of responses. Overall, similar elevations in frequency and VT were observed across groups during Max relative to BL. However, DHT appeared to blunt the overall ventilatory response to Max challenge relative to Sham groups **(C)** All data assessed using one-way ANOVA and Bonferroni *post hoc* tests where appropriate. *p < 0.05.

In addition to exploring changes in raw and weight adjusted ventilatory measures, we assessed frequency, VT and VE data as a %change from BL conditions to gauge the magnitude of respiratory responses during Max challenge. There was no overall effect of treatment on the magnitude of frequency or VT change with Max challenge (*p* = 0.15 and *p* = 0.74 respectively). All groups showed similar magnitude of frequency (Figure 4A) and VT responses relative to BL (Figure 4B). Modest reductions in frequency and VT response in the DHT group (Figures 4A,B) appeared to contribute to a treatment effect in the magnitude of VE response to Max challenge (*p* = 0.02; Figure 4C). SCI-DHT rats showed a statistically lower VE response to Max challenge compared with uninjured Sham rats (*p* = 0.04). The SCI-placebo group also appeared to have a reduced VE response compared with Sham rats, but this difference did not reach a level of significance (*p* = 0.099; Figure 4C).

**FIGURE 5:**
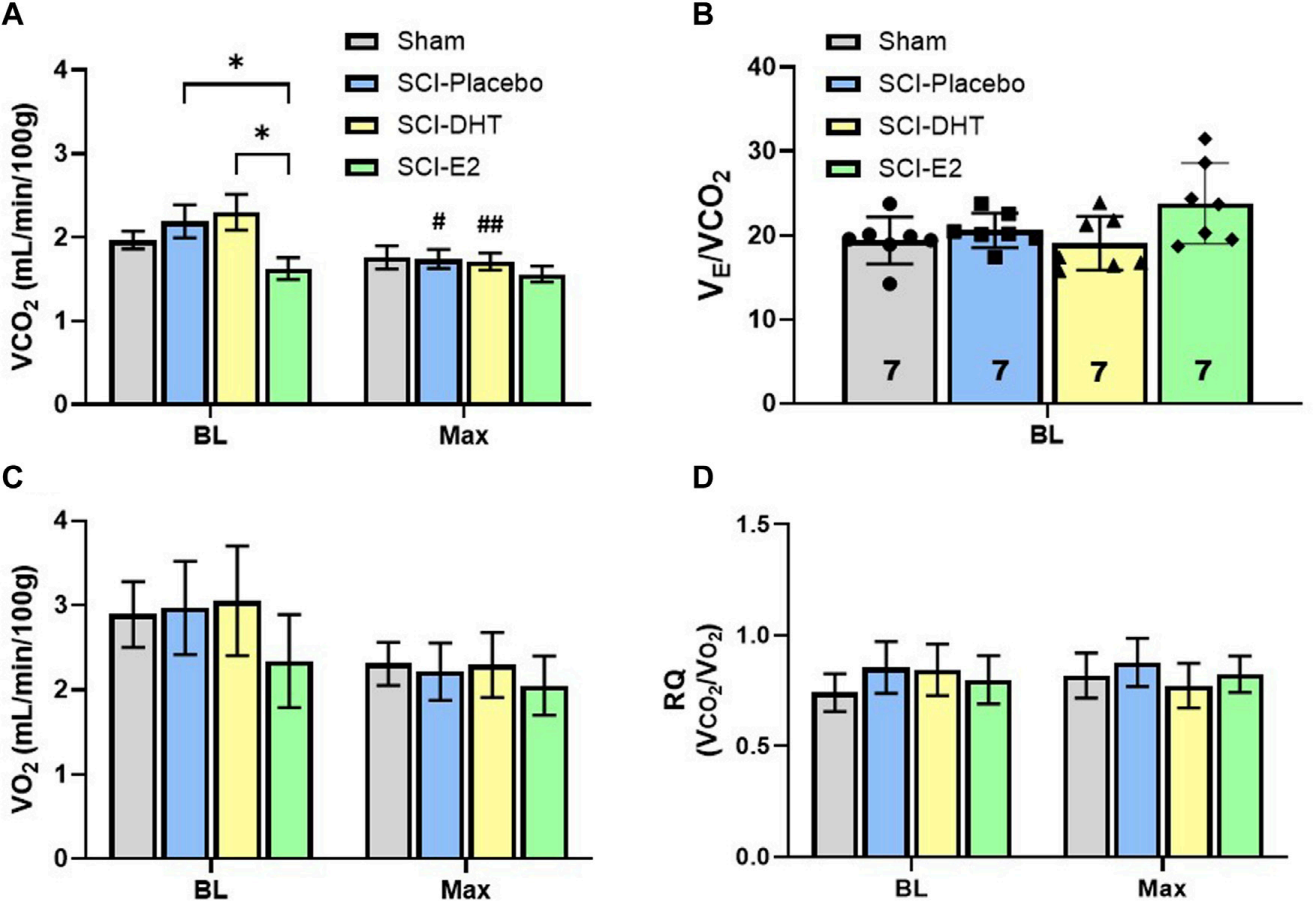
Estrogen supplementation reduced basal metabolism following C2-hemisection. VCO_2_ was quantified during BL and Max respiratory challenge to determine if steroid hormone supplementation influenced CO_2_ production **(A)**. Two-Way ANOVA revealed a significant condition effect (*p* < 0.001) and *post hoc* analysis revealed that E2 supplementation resulted in significantly lower VCO_2_ production during normoxic BL conditions compared to SCI-placebo or SCI-DHT treated rats. No change in VCO_2_ was seen during respiratory challenge in uninjured Sham rats or rats receiving E2 supplementation compared to BL. However, SCI-placebo and SCI-DHT treated rats showed significant reductions in VCO_2_ during respiratory challenge compared to BL. In **(B)**, we show that despite differences in BL VCO_2_ production, VE was similar between groups when we control for VCO_2,_ though a modest trend towards higher BL VE in the SCI-E2 groups was observed (One-Way ANOVA; *p* = 0.056). No differences in oxygen consumption (VO_2_) were observed during BL or Max (Two-Way ANOVA; **(C)**). Finally, we calculated the respiratory quotient (RQ; VCO_2_/VO_2_) to determine if changes in macronutrient metabolism may be present (Two-Way ANOVA; **(D)**). RQ was stable and similar across experimental groups and respiratory conditions. *:*p* < 0.05; #*p* < 0.05 from BL; ##*p* < 0.01 from BL.

Our plethysmography system enables sampling of expired gas concentrations, and these were used to measure carbon dioxide production (VCO_2_) and oxygen consumption (VO_2_); indicators of metabolic rate. Figure 5A shows differences in VCO_2_ during BL and Max respiratory challenge. A significant effect of respiratory gas condition on VCO_2_ was observed with Two-Way ANOVA (*p* < 0.001, Figure 5A), and *post hoc* analyses revealed both within-group and between group differences in VCO_2_. During BL, SCI rats receiving E2 supplementation exhibited similar VCO_2_ (1.63 mL/min/100 g) as Sham rats (1.97 mL/min/100 g; *p* = 0.61), but significantly reduced VCO_2_ relative to SCI-DHT (2.30 mL/min/100 g; *p* = 0.02) and SCI-placebo groups (2.19 mL/min/100 g; *p* = 0.05; Figure 5A). This treatment effect disappeared during Max challenge, as all groups showed similar VCO_2_. Interestingly, the SCI-DHT (*p* = 0.005) and SCI-placebo rats (*p* = 0.02) significantly decreased their VCO_2_ during Max challenge compared with BL (Figure 5A). Collectively, these data suggest that E2 supplementation normalized metabolism across respiratory conditions after C2Hx. However, when we analysed the impact of VCO_2_ on VE, no additional group differences were revealed. For example, when we compared ventilatory efficiency (VE/VCO_2_) across groups during BL, a treatment effect of E2 appeared to emerge, but fell just short of significance (*p* = 0.06; Figure 5B). There were no differences in VO_2_ across groups during BL or with Max respiratory challenge (*p* > .99; Figure 5C). Finally, we calculated the respiratory quotient (RQ; VCO_2_/VO_2_) to determine if changes in macronutrient metabolism may be present (Figure 5D). Although Sham rats tended to display lower BL RQ values (0.74 ± 0.10) relative to all other groups, and DHT rats tended to have lower RQ during Max (0.77 ± 0.09) the differences did not reach significance (treatment effect *p* = 0.93; condition effect *p* = 0.52; Figure 5D). RQ was stable and similar across experimental groups and respiratory conditions.

### 3.3 DHT and estradiol enhance hypoxic neural responses and phrenic neuroplasticity

**FIGURE 6:**
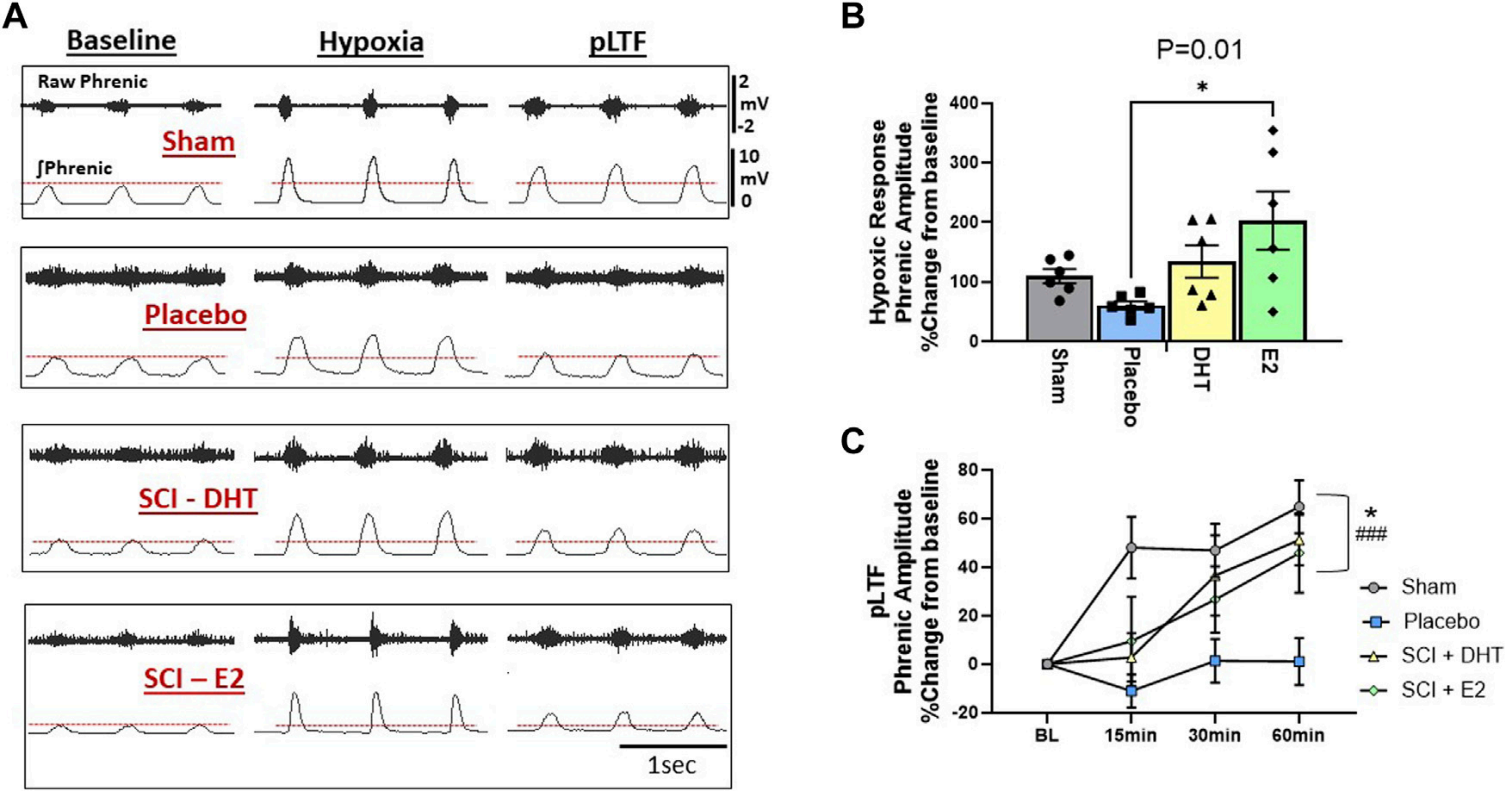
Hormone supplements enhance neural response to hypoxia and the magnitude of respiratory neuroplasticity. We assessed the ipsilateral phrenic neural response to hypoxia and the magnitude of acute intermittent hypoxia (AIH)-induced phrenic long-term facilitation (pLTF) in the ipsilateral phrenic nerve 2 weeks following C2Hx. Panel **(A)** provides representative examples of raw and integrated ipsilateral phrenic nerve signals from each experimental group during BL, hypoxia, and 60-min following hypoxia for quantification of pLTF. The red dotted lines denote BL ipsilateral phrenic amplitude for comparison across time. One-Way ANOVA revealed differences in the neural response to hypoxia **(B)**. Rats receiving E2 supplementation showed a significantly larger neural response to hypoxia compared to the SCI-placebo group. Magnitude of pLTF is revealed as a significant increase in phrenic amplitude relative to BL following AIH **(C)**. Two-way ANOVA with repeated measures revealed significant main effects of time (*p* < 0.0001), treatment (*p* < 0.02), and a significant time × treatment interaction (*p* < 0.005). Sham, SCI-DHT, and SCI-E2 rats all showed significant elevations in phrenic amplitude relative to BL by 60 min post AIH (i.e., pLTF). In addition, the magnitude of pLTF in rats receiving E2 and DHT supplementation was similar to uninjured Sham rats. Sham (*p* = 0.0005), SCI-DHT (*p* = 0.009), and SCI-E2 rats (*p* = 0.024) showed significantly higher pLTF 60 min post-AIH compared to SCI-placebo rats. ###:*p* < 0.001 from BL; *:*p* < 0.05 from C2Hx.

To determine the impact of hormone supplementation on ipsilateral phrenic motor output following C2Hx, we quantified the magnitude of phrenic neural output in response to AIH and for 60 min following AIH to quantify whether a neuroplastic response, (i.e., phrenic long-term facilitation [pLTF]), was induced. One rat from each treatment group was removed from neurophysiological comparisons due to either surgical complications resulting in damaged ipsilateral phrenic nerves, or because blood gas values fell outside of predetermined ranges (see below). Representative examples of raw and integrated phrenic neural signals during BL, hypoxia, and 60 min post-hypoxia (i.e., pLTF) are shown in Figure 6A.

Group comparisons of the phrenic neural responses to hypoxia are presented in Figure 6B. There was an overall impact of treatment on ipsilateral phrenic output with hypoxia (One-way ANOVA; *p* = 0.02; Figure 6B). When compared to Sham rats alone, SCI-placebo rats showed an expected reduction in ipsilateral phrenic neural output in response to hypoxia (*p* = 0.005; unpaired, two-tailed *t*-test; not shown). Post-hoc analyses revealed that the SCI-E2 group showed larger ipsilateral neural responses to hypoxia than untreated SCI-placebo rats (*p* = 0.01; Figure 6B). DHT (134% increase) also appeared to increase hypoxic responses relative to Placebo (61% increase), but this did not reach significance (*p* = 0.29). SCI-DHT (*p* = 0.92) and SCI-E2 (*p* = 0.63) groups had similar magnitudes of hypoxic response as sham control rats (Figure 6B).

Hormone supplementation had a significant impact on the expression of ipsilateral pLTF 2 weeks post-SCI. Two-way ANOVA with repeated measures revealed significant main effects of time (*p* < 0.0001) and treatment (*p* = 0.02) and a significant time × treatment interaction (*p* = 0.005). Sham and SCI-DHT groups showed significantly elevated phrenic neural activity relative to BL at all post-AIH time points (*p* ≤ 0.03 for each; significance markers not shown; Figure 6C). Rats in the SCI-E2 group displayed a steady increase in phrenic nerve amplitude relative to BL following AIH that became significant 60 min post-AIH (*p* = 0.001). Collectively, AIH induced pLTF was revealed by 60 min post-AIH in Sham, SCI-DHT, and SCI-E2 groups (all *p* ≤ 0.001); untreated rats receiving placebo pellets did not express pLTF (*p* > 0.99 relative to BL; Figure 6C). In addition, the magnitude of pLTF in rats receiving E2 and DHT supplementation was similar to uninjured Sham rats. Sham (*p* = 0.0005), DHT (*p* = 0.009), and E2 rats (*p* = 0.024) showed significantly higher pLTF 60 min post-AIH compared to rats with SCI alone (Figure 6C).

Table 2 provides the blood gas analyses collected during phrenic nerve recording experiments. All groups were maintained within *de novo* parameters to ensure valid conditions for comparison. These parameters included: core body temperature of 37°C ± 1°C throughout the experiment, PaCO_2_ ± 1.5 mmHg from BL measures across experimental conditions, PaO_2_ above 150 mmHg except during AIH (see Methods), standard base excess (SBE) was maintained ±3.0 mEq/L, and MAP was stable, not fluctuating more than 30 mmHg below BL measures in the post-AIH period. One interesting difference among groups: rats treated with E2 started with elevated PaCO_2_ relative to sham and the other SCI groups. This difference between SCI rats was maintained 60 min post-AIH (Table 2).

**TABLE 2:** Physiological parameters during phrenic nerve recordings.

Condition	Treatment	Temp. (°C)	pH	PACO_2_ (mmHg)	PAO_2_ (mmHg)	SBE (mequiv l−1)	MAP (mmHg)
Baseline	Sham	36.8 ± 0.2	7.409 ± 0.01^++^	37.4 ± 0.8^+++^	231 ± 8	−1.3 ± 0.4	119 ± 3
	Placebo	36.5 ± 0.1	7.431 ± 0.01^+++^	37.0 ± 1.6^+++^	232 ± 7	−0.1 ± 0.6	97 ± 8
	SCI-DHT	36.3 ± 0.2	7.441 ± 0.01^+++^	39.8 ± 1.3^++^	235 ± 8	2.2 ± 0.6^##^	105 ± 5
	SCI-E2	36.7 ± 0.2	7.346 ± 0.01	48.8 ± 2.7	239 ± 3	−0.2 ± 0.7	96 ± 7
Hypoxia	Sham	36.8 ± 0.2	7.416 ± 0.01^++^	37.7 ± 0.6^++^	41.6 ± 1.8	−0.6 ± 0.2	119 ± 6
	Placebo	36.7 ± 0.1	7.433 ± 0.01^+++^	37.5 ± 1.6^++^	47.1 ± 2.9	0.4 ± 0.6	86 ± 11
	SCI-DHT	36.5 ± 0.3	7.405 ± 0.01^++^	39.0 ± 1.1^++^	42 ± 1.8	−0.8 ± 0.4	95 ± 7
	SCI-E2	36.8 ± 0.1	7.339 ± 0.01	48.5 ± 2.8	44 ± 2	−0.7 ± 0.6	108 ± 8
60 min	Sham	36.9 ± 0.2	7.404 ± 0.01	39.0 ± 1.0^++^	216 ± 8	−0.6 ± 0.3	109 ± 6
	Placebo	36.7 ± 0.1	7.439 ± 0.01^++^	37.1 ± 1.7^++^	205 ± 33	0.5 ± 0.7	109 ± 6
	SCI-DHT	36.5 ± 0.2	7.430 ± 0.01^++^	39.9 ± 1.3^+^	233 ± 6	1.6 ± 0.6	111 ± 6
	SCI-E2	36.8 ± 0.2	7.359 ± 0.01	49.4 ± 2.9	231 ± 7	1.1 ± 0.6	92 ± 6

Data represent One-Way ANOVA between experimental groups within each condition. No statistical differences were noted in temperature, PAO_2_, or MAP in any conditions. SCI-E2 rats showed statistically lower pH compared to all other groups during BL and hypoxia, and lower than SCI-placebo and SCI-DHT groups at 60 min post-AIH. SCI- E2 rats also showed higher PACO_2_ values compared to all other groups in all conditions. +: *p* < 0.05, ++*p* < 0.01, +++*p* < 0.001 from SCI-E2. ##*p* < 0.01 from Sham.

## 4 Discussion

In addition to the loss of motor and sensory function below the injury, a secondary consequence of SCI is a reduction in circulating sex steroid hormones, which are key to normal function across physiological systems. These hormones are also critically important to neuroplasticity. Reduced circulating steroid hormones may impede our ability to induce beneficial plasticity to optimize motor recovery following SCI. This study was designed to determine whether hormone supplementation could improve overall respiratory function and the expression of AIH-induced respiratory motor plasticity in a model of sub-acute cervical SCI. Adjuvant hormonal supplementation has been used in SCI studies in both humans and rodents with positive results. Both testosterone and E2 are effective in protecting against cell death (Pike, 2001; Yune et al., 2004), promoting functional recovery in motor function following SCI (Sribnick et al., 2003; Sribnick et al., 2005; Samantaray et al., 2010; Sribnick et al., 2010), and stimulating axonal growth in neurons (Kujawa et al., 1989; Islamov et al., 2003). However, whether hormonal supplementation would influence respiratory recovery or the induction of respiratory neuroplasticity following cervical SCI was unknown.

Our primary findings were: 1) C2Hx significantly reduced serum testosterone levels for at least 2 weeks post-injury; 2) E2 supplementation starting 1 week post cervical-SCI, resulted in reduced respiratory frequency and increased VT generation during respiratory challenge when compared with rats receiving no hormonal treatments. These changes reflected a return to normal breathing patterns similar to uninjured rats, though the net resulting VE was similar to other treatment groups; 3) E2 supplementation appeared to beneficially regulate baseline metabolism in sub-acute cervical SCI; and 4) Supplementation with either E2 or DHT was sufficient to restore the expression of AIH-induced pLTF, a well characterized form of respiratory neuroplasticity, 2 weeks post-SCI. Collectively, our findings demonstrate that E2 supplementation can positively impact respiratory function and supplementation with E2 or DHT are sufficient to restore respiratory neural plasticity in male rats following SCI, providing promising avenues for further research and potential therapeutic interventions to maximize respiratory motor recovery.

The C2Hx injury model is an important pre-clinical cervical SCI model often used to study recovery of respiratory function. C2Hx separates neural connections between brain stem respiratory centers and the phrenic motor neurons that innervate the diaphragm muscle, resulting in partial paralysis of the ipsilateral diaphragm. The subsequent loss of full respiratory motor capacity induces long-lasting respiratory impairments that are similar to those observed in human patients with cervical spinal cord injury. What was unknown until now, was whether the C2Hx model replicated other secondary consequences of human SCI; specifically, the significant reduction in serum steroid hormone levels seen in nearly all men with SCI (Clark et al., 2008; Yarrow et al., 2020). Our data demonstrate that C2Hx causes a reduction in serum testosterone (the primary male sex steroid hormone) for at least 2 weeks post injury. This is the first demonstration that C2Hx directly reduces circulating sex hormone levels and is consistent with clinical findings indicating low testosterone levels in males for the first 12 months post-injury.

Testosterone may have direct effects on neural function through its activation of androgen receptors (AR) and subsequent AR signaling cascades. However, testosterone can also be enzymatically metabolized into dihydrotestosterone (DHT) or 17β-estradiol (E2), enabling diverse physiological effects through divergent signaling pathways (Figure 1). Aromatization of testosterone to E2 would precede activation of local estrogen receptors, while DHT, converted from testosterone via 5α-reductase, would exert its effects through AR signaling mechanisms similar to testosterone. For this study, we chose to supplement with these active testosterone metabolites as a first step in discerning which cellular mechanisms may induce functional changes. We first used whole-body plethysmography to explore the overall impact of C2Hx, and the subsequent effects of hormone supplementation, on respiratory function 2 weeks post injury. Rats receiving C2Hx alone (the SCI-placebo group) showed a stereotypical change in respiratory pattern consisting of increased respiratory frequency and diminished VT production that was revealed primarily during respiratory challenge. This change in respiratory pattern after injury permits maintenance of overall VE. Accordingly, VE was similar between all experimental groups (and similar to uninjured Sham control rats) during BL and Max respiratory challenge conditions. However, rats receiving E2 supplementation reverted back to a respiratory pattern (i.e., slower frequency and larger VT) similar to Sham rats in both respiratory conditions. The ability to restore normal respiratory patterns 2 weeks post-SCI is significant, as it may reflect a strengthening of residual neural pathways to ipsilateral phrenic motor neurons, or other respiratory motor pools, and may indicate an overall increase in respiratory capacity. Unveiling that possibility would likely require a more severe, or prolonged respiratory challenge in order to clearly assess the ability of E2 supplemented rats to respond to increased respiratory demand.

We also used our plethysmography data to inspect metabolic differences between groups. Studies in humans and rats indicate that SCI induces complex, temporal changes to basal metabolic rate, and metabolism significantly impacts ventilation (Chiu and Lee, 1985; Mortola and Maskrey, 2011). It is well known that humans and rats will undergo a significant decline in basal metabolism overtime following injury due to chronic loss of skeletal muscle mass (Chiu and Lee, 1985; Smith and Yarar-Fisher, 2016). However, the acute and sub-acute periods following injury can be associated with a hypermetabolic state, catabolism, and accelerated nitrogen loss (Kinney et al., 1970; Rodriguez et al., 1997). Here, C2Hx rats showed a modest increase in carbon dioxide production (VCO_2_) and stable levels of oxygen consumption (VO_2_) under BL, room air conditions 2 weeks post-SCI. These findings suggest that in the subacute recovery period, overall basal metabolism may be heightened. These finding are consistent with recent rat SCI studies using a cervical contusion model. Chiu and Lee (2021) showed that rats with severe lateralized contusion injuries at the C3-C4 cervical segment had similar VO_2_ as uninjured rats under room-air conditions, but gradually elevating VCO_2_ values by 2 weeks post-injury. As rats continued to age, basal metabolism significantly dropped as evidenced by depressed VO_2_ and VCO_2_ measures 8-week post-injury, consistent with human studies (Chiu and Lee, 1985). Here, E2 supplementation following SCI had a significant effect on BL metabolism, bringing BL VCO_2_ values back to control levels and increasing VE/VCO_2_ relative to other experimental groups (though falling just short of statistical significance). E2, the most prevalent and neuroactive form of estrogen, has been shown to protect the nervous system by reducing inflammation, attenuating apoptotic cell death, reducing lesion size, increasing white matter sparing, and altering cytokine release and astroglial responses post-injury (Yune et al., 2004; Sribnick et al., 2005; Sribnick et al., 2010; Samantaray et al., 2016). In addition, E2 maintains skeletal muscle size and strength in males and females. Our method of using subcutaneous pellets did not permit the localization of E2 effects in this study, so a combination of these factors may have contributed to the improved respiratory function and stabilized metabolism in our E2 rats. In addition, since chronic SCI in rats and humans is associated with reduced basal metabolism over time, assessing whether E2 supplementation blunts the chronic reduction in basal metabolism could have significant clinical utility for improving motor function.

A principal goal for rehabilitation professionals is to maximize function in patients with SCI by inducing targeted neuroplasticity. The respiratory motor system is capable of robust neuroplasticity in response to AIH, resulting in the progressive augmentation of phrenic motor output known as phrenic long-term facilitation (pLTF). With reduced bulbospinal and neuromodulatory input to ipsilateral phrenic motor neurons following C2Hx, AIH-induced pLTF is usually not inducible 2 weeks post-hemisection (Golder and Mitchell, 2005). To our surprise, a 7 days supplement of either DHT or E2 was sufficient to restore ipsilateral pLTF at this subacute post-injury time point. We hypothesized that E2 supplementation would restore pLTF based on prior studies indicating testosterone, aromatized to E2, was sufficient to enable AIH-induced pLTF in male rats when serum testosterone levels were reduced following castration. We found that testosterone levels were reduced with SCI, so we predicted that E2 would have similar facilitating impacts in our sub-acute SCI rats. Prior reports suggest that the effects of estrogen on respiratory function likely occur in the CNS vs peripheral sites of action like the carotid chemoreceptors (Behan et al., 2003; Behan and Kinkead, 2011). These peripheral sites are more sensitive to progesterone, another abundant female steroid hormone (Tatsumi et al., 1985; Dempsey et al., 1986; Kimura et al., 1986; Kimura et al., 1988). The blood gas data from our neurophysiological studies indicated that rats with E2 supplementation displayed elevated blood CO_2_ levels under control conditions, suggesting that E2 supplementation may have impacted CO_2_ sensing or sensitivity in this experimental preparation. How this change in CO_2_ may have influenced the changes in AIH-induced pLTF following C2Hx is a topic for future investigation. A recent report suggested that elevated PaCO_2_ during BL phrenic recording conditions may reduce the magnitude of AIH-induced pLTF (Perim et al., 2021). Thus our findings may, in fact, underestimate the impact of supplemental E2 on respiratory neuroplasticity given the elevated BL PaCO_2_ levels observed. However, in our study (different than (Perim et al., 2021)) rats underwent an apneic/recruitment threshold test at the start of phrenic recordings to normalize respiratory stimulation across experimental groups. Therefore, despite elevated PaCO_2_, rats in the E2 group should have received similar levels of chemostimulation throughout the experiment. Regardless, our findings support the premise that E2 supplementation has beneficial impacts on expression of respiratory neuroplasticity in sub-acute SCI.

In prior work, DHT supplementation was unable to restore pLTF when circulating testosterone levels were low (Zabka et al., 2006). The fact that DHT was sufficient to restore pLTF under conditions of sub-acute SCI suggests a unique mechanism of action that was specific to the pathophysiology of SCI, versus a reduction in testosterone levels *per se*. In pre-clinical models of SCI, testosterone and DHT appear to exert therapeutic effects mostly through the maintenance of muscle mass and body composition (Gregory et al., 2003). It is feasible that DHT could have had similar effects on our SCI rats, but multiple points of evidence suggest otherwise. For example, improved respiratory muscle function relative to untreated SCI rats should have resulted in better overall respiratory outcomes measured with plethysmography. Rather, rats receiving DHT had similar ventilation as placebo rats. Also, our metabolic findings indicate that DHT supplementation had little impact on basal metabolic rate relative to placebo treated rats. If DHT supplementation was significantly increasing (or maintaining) skeletal muscle mass, then an increase in basal metabolism would be anticipated. Prior evidence in rodent SCI models indicates that DHT supplementation may act to maintain dendritic length in motor neurons (Byers et al., 2012). C2-hemisection imparts significant anatomical changes to the local phrenic motor circuitry (Lane et al., 2009), and maintaining dendritic structure could provide a substrate to enable AIH-induced pLTF via strengthening of direct bulbospinal connectivity, or strengthening of multi-synaptic connections acting through cervical interneurons. DHT may also directly affect phrenic motor neuron function, as ARs have been shown in phrenic motor neurons (Behan and Thomas, 2005). However, the impact of SCI on AR expression in the respiratory motor system is unknown.

Respiratory complications are among the top causes of reduced quality of life and mortality in individuals with SCI (Berlowitz et al., 2016; Savic et al., 2017; Zakrasek et al., 2017). Steroidal sex hormones, such as testosterone and estrogen, are important regulators of respiratory function, necessary to permit neuroplasticity in the respiratory motor system, and beneficially impact functional recovery following SCI. Our data align with these findings and reveal positive effects of E2 and DHT supplementation on ventilation and neuroplasticity 2 weeks post-C2-hemisection. Hormone-based interventions may hold promise to strengthen respiratory function in models of cervical SCI and have the potential to translate to clinical application as part of a holistic approach to maximizing function.

## Data Availability

The raw data supporting the conclusion of this article will be made available by the authors, without undue reservation.
